# Contributors to the 2025 *RSC Chemical Biology* Emerging Investigators Collection

**DOI:** 10.1039/d5cb90052b

**Published:** 2025-12-16

## Abstract

This article profiles the early career researchers whose work features in the *RSC Chemical Biology* Emerging Investigators Collection 2025.
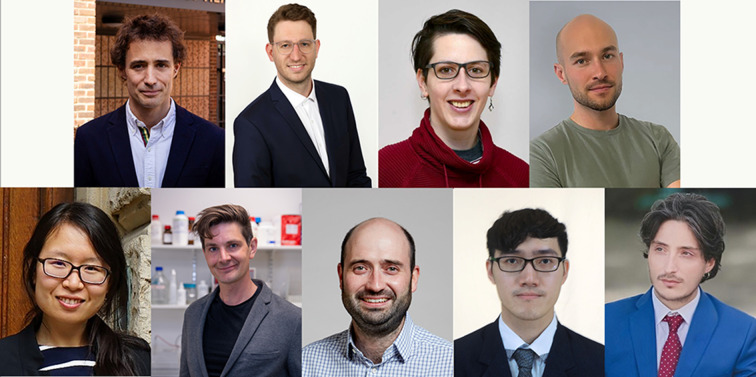


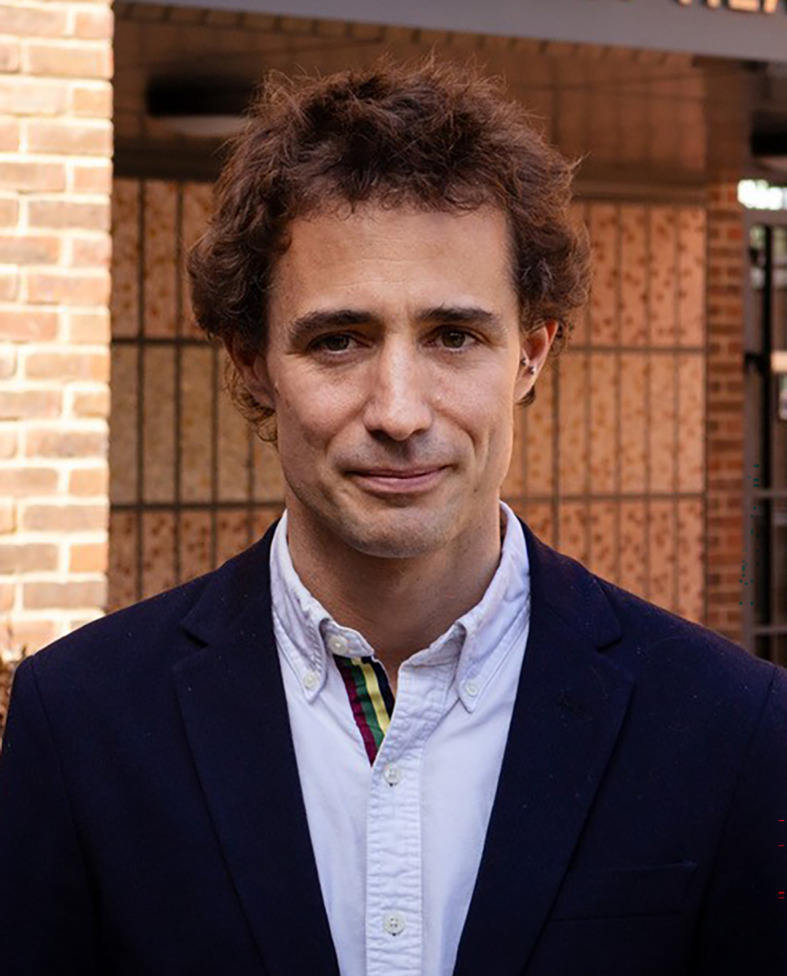
Pietro Sormanni is a Royal Society University Research Fellow leading a group at the University of Cambridge and the recipient of the 2025 Norman Heatley Award from the Royal Society of Chemistry. His research lies at the interface of computational method development and experiments. The group's main goal is to create computational antibody-design technologies that transform antibody discovery and engineering. Their work, also through industrial and academic partnerships, already shows these methods can streamline antibody development and deliver faster, cheaper alternatives to traditional routes. Previously, Pietro was a Borysiewicz Biomedical Sciences Fellow at Cambridge. He holds a PhD in Chemistry and an MSc in Theoretical Physics.

His contribution to the 2025 *RSC Chemical Biology* Emerging Investigators collection can be read at https://doi.org/10.1039/D5CB00079C.


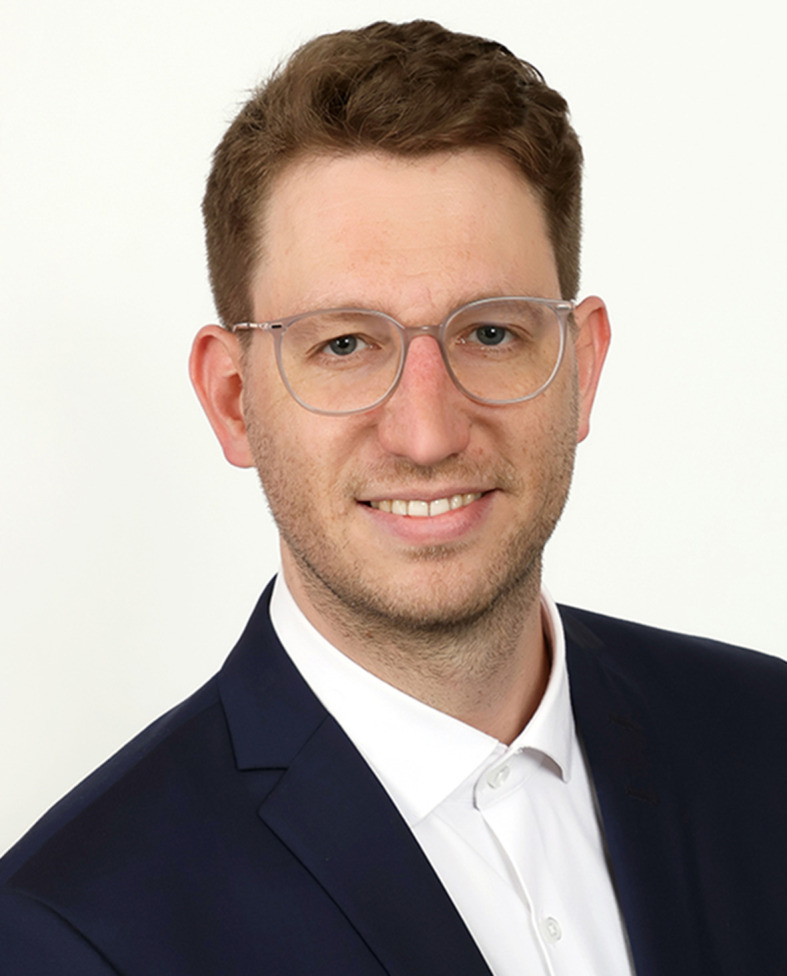
Nicolas Cornelissen is a research group leader at the University of Münster. Born in Germany and raised in Portugal, he studied chemistry at the University of Münster, including a research stay at the Universidade Federal do Rio de Janeiro, Brazil. He obtained his PhD with Prof. Andrea Rentmeister on enzymatic cascades for selective alkylation using methyltransferases. His research interests include the chemo-enzymatic synthesis of modified nucleotides and the generation of modified nucleic acids for the study of biological systems and as a therapeutic modality.

His contribution to the 2025 *RSC Chemical Biology* Emerging Investigators collection can be read at https://doi.org/10.1039/D5CB00108K.


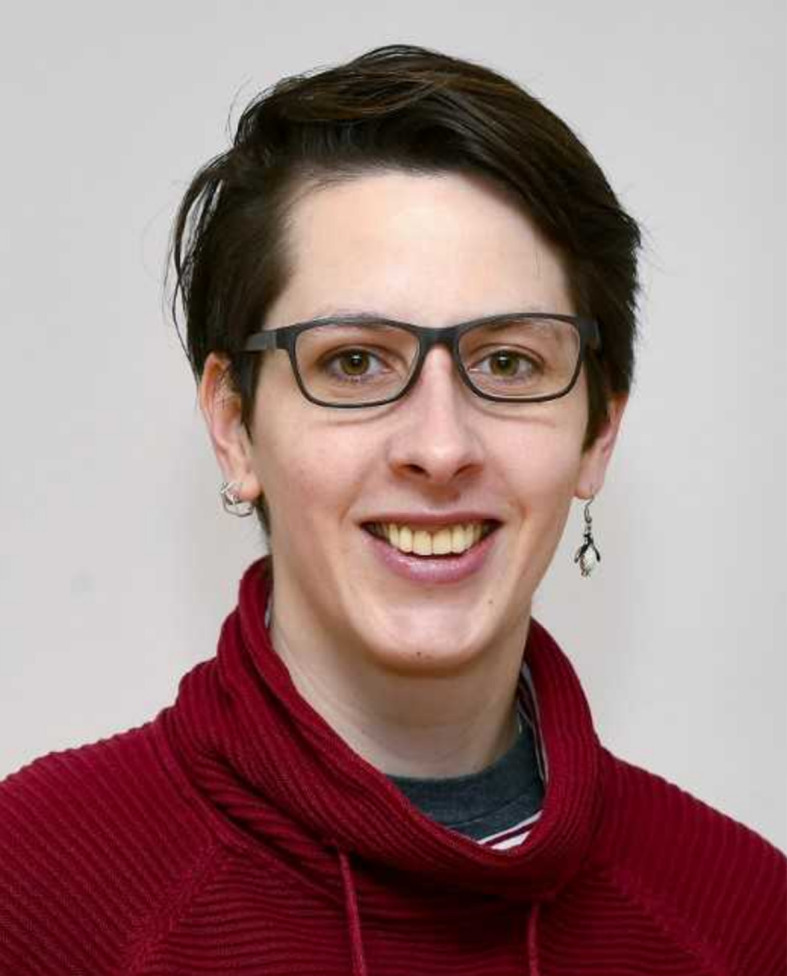
Nadja A. Simeth pursued her doctorate studies with Burkhard König at the University of Regensburg, Germany, as a fellow of the Studienstiftung des Deutschen Volkes. She later joined the group of Ben L. Feringa at the University of Groningen as a postdoc supported by a Feodor-Lynen Fellowship of the Humboldt Foundation. In autumn 2021, she was appointed as assistant professor at the University of Göttingen and promoted to associate professor in April 2025. She is interested in the design of smart fluorophores, light-controlled pharmacophores, and responsive biomolecules.

Her contribution to the 2025 *RSC Chemical Biology* Emerging Investigators collection can be read at https://doi.org/10.1039/D5CB00124B.


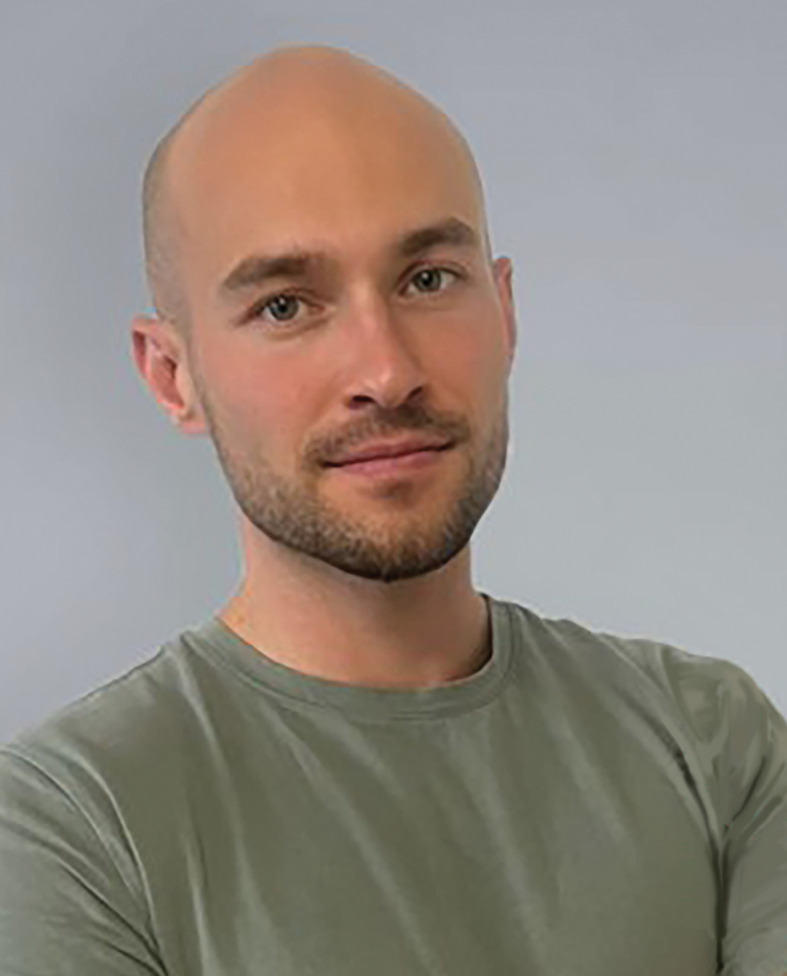
Václav Němec is a medicinal chemist and team leader at the Structural Genomics Consortium (SGC). He completed his MSc and PhD in Organic Chemistry at Masaryk University, Czech Republic, where he developed highly selective kinase inhibitors (CLKs, HIPKs, ALK1/2, CK1). During his PhD, he undertook a research stay with Prof. David Procter at the University of Manchester, investigating SmI_2_ radical cascades for the synthesis of a complex natural product. After finishing his PhD in 2020, he joined the SGC, where he leads medicinal chemistry projects at the interface of academia and industry. His contributions have resulted in first-in-class TRIM7 ligands, high-quality chemical probes, PROTAC degraders and establishment of a chemogenomic library.

His contribution to the 2025 *RSC Chemical Biology* Emerging Investigators collection can be read at https://doi.org/10.1039/D5CB00109A.


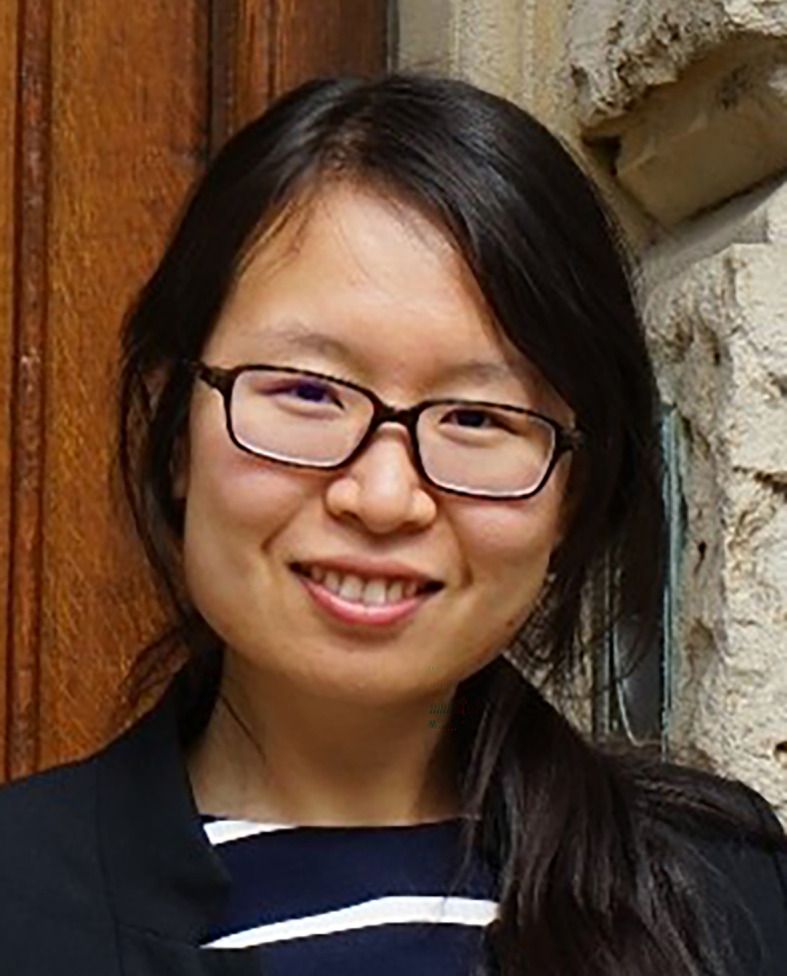
Shiqi Wang received her bachelor's and master's degrees from Tsinghua University China in 2012 and 2014 respectively. Then she moved to Imperial College London as a Marie Curie Early-Stage Researcher in the department of chemical engineering, where she got her PhD degree in 2018. Afterwards, she joined the Faculty of Pharmacy, University of Helsinki as a postdoctoral researcher. In 2024 she started her own research group at the same Faculty. Her group aims to understand how nanoparticles deliver biological drugs inside cells and to use this understanding to develop new nanomedicine for novel biotherapeutics.

Her contribution to the 2025 *RSC Chemical Biology* Emerging Investigators collection can be read at https://doi.org/10.1039/D5CB00104H.


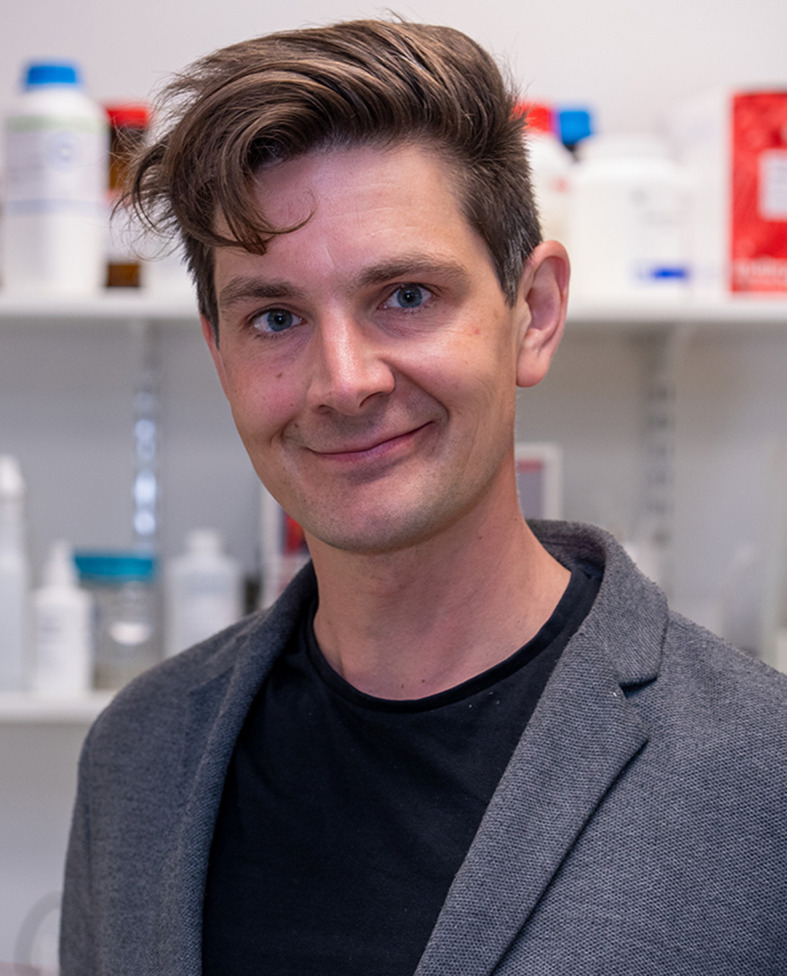
Alex Taylor is a Wellcome Trust and Royal Society Sir Henry Dale Fellow in the Department of Chemistry at King's College London. He trained as a molecular immunologist in the Randall Division, KCL and moved into synthetic biology and nucleic acid chemistry as a postdoc with Philipp Holliger at the MRC Laboratory of Molecular Biology. In 2020, Alex established his group at the University of Cambridge and in 2024 returned to KCL, accepting a proleptic Lectureship in Chemical Biology. His research explores the diversity of synthetic chemical structures and novel biochemical functions accessible through test-tube evolution of artificial xeno nucleic acid (XNA) analogues and seeks to engineer XNA tools and technologies for potential applications in precision medicine.

His contribution to the 2025 *RSC Chemical Biology* Emerging Investigators collection can be read at https://doi.org/10.1039/D5CB00182J.


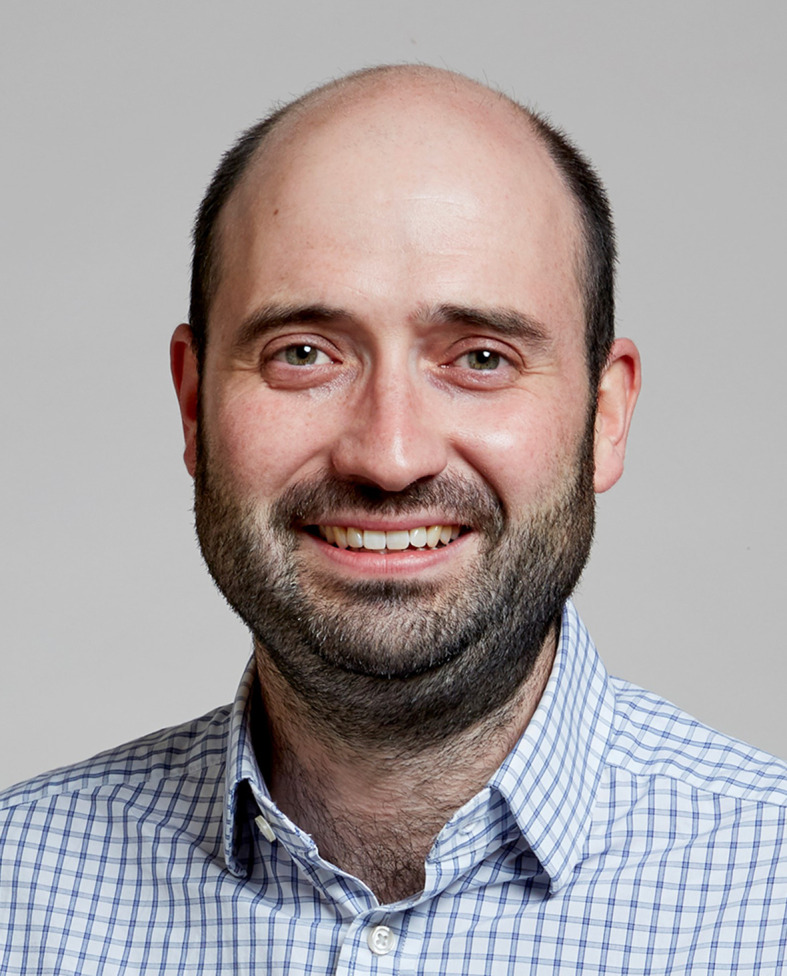
Tom McAllister is a Royal Society University Research Fellow in the School of Natural and Environmental Sciences at Newcastle University. He completed his undergraduate and doctoral studies at the University of Leeds, post-doctoral research at Leeds and the University of Oxford and took up his current post in 2022. He has focussed on protein post-translational modifications throughout his career, and the McAllister group now develop new tools and approaches to investigate the biological roles of protein glycosylation using a combination of molecular biology, peptide and carbohydrate chemistry.

His contribution to the 2025 *RSC Chemical Biology* Emerging Investigators collection can be read at https://doi.org/10.1039/D5CB00076A.


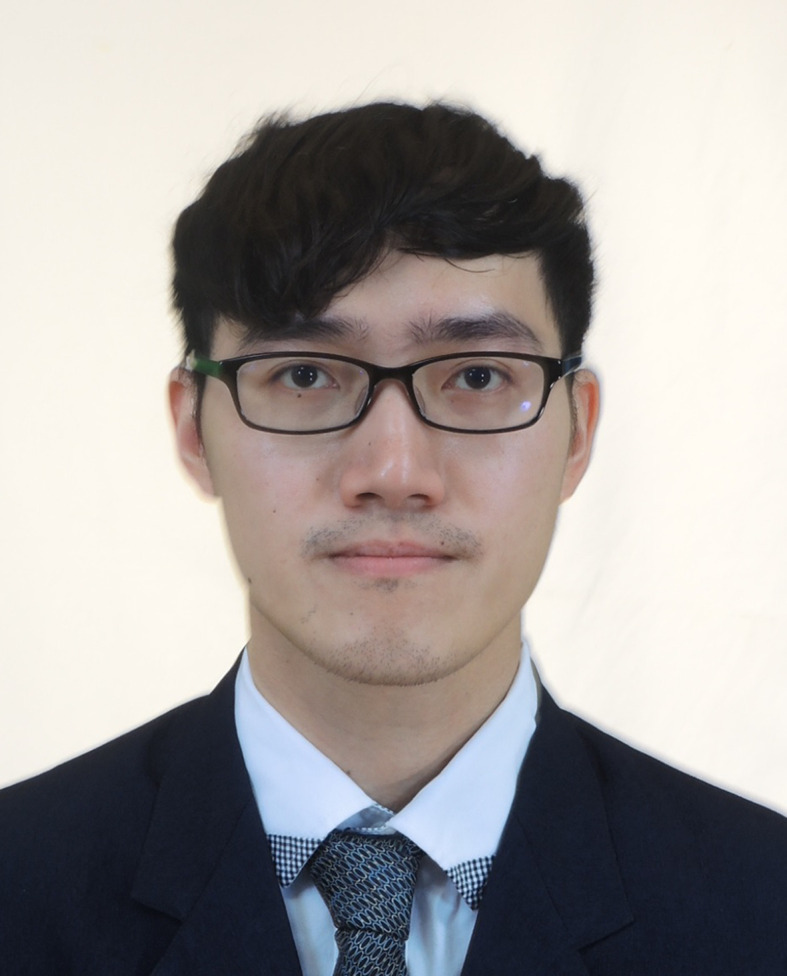
Chin-Soon Phan was trained in marine natural products chemistry and obtained his PhD from UMS, Malaysia. He then carried out research on natural products biosynthesis at the University of Western Australia (Yit-Heng Chooi) as visiting fellow, Hokkaido University (Tatsufumi Okino and Toshiyuki Wakimoto) as JSPS postdoc fellow and National University of Singapore (Brandon I. Morinaka) as postdoc researcher. In December 2023, he started his independent career at the Latvian Institute of Organic Synthesis, focusing on cross-linking enzymes involved in the RiPP biosynthesis.

His contribution to the 2025 *RSC Chemical Biology* Emerging Investigators collection can be read at https://doi.org/10.1039/D5CB00153F.


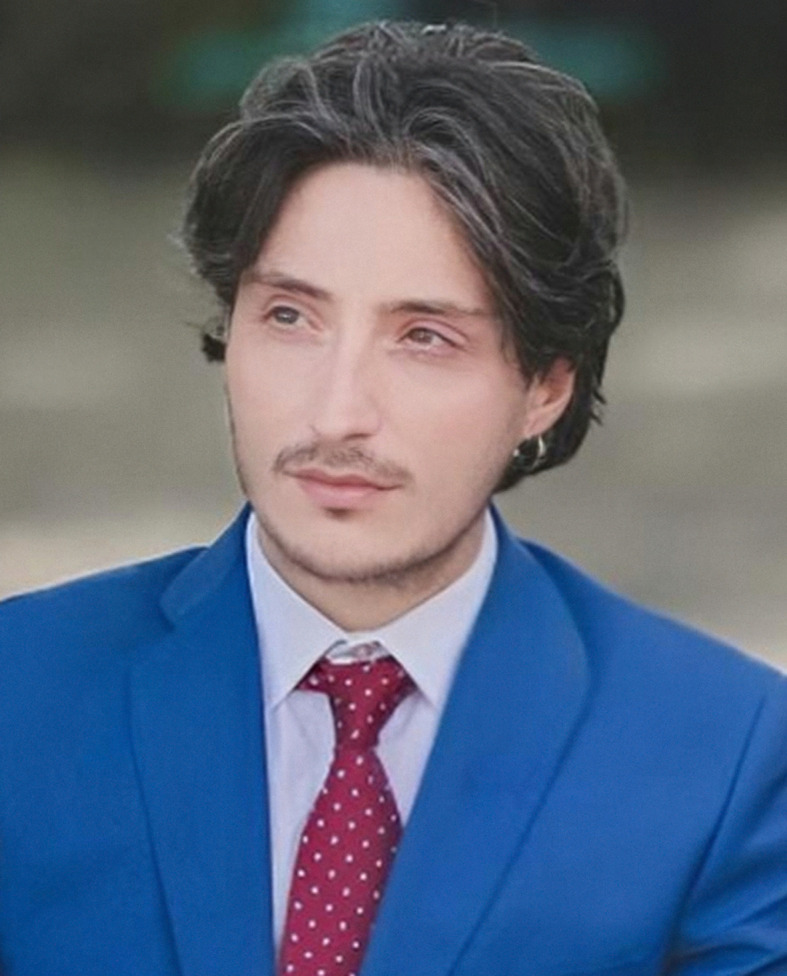
Enrico Cadoni is currently an MSCA Fellow at Imperial College London. He earned a single-cycle degree (*cum laude*) in Pharmaceutical Sciences from Sapienza University of Rome and a PhD in Chemistry from Ghent University, where he studied, among other things, the development of chemical tools to manipulate nucleic acid secondary structures (G-quadruplexes and I-motifs). Following a postdoctoral appointment in Ghent funded by an FWO Postdoctoral Fellowship, during which he investigated the covalent stabilization of nucleic acid aptamers, his current research focuses on using photochemical approaches to control nucleic acid secondary structures in living cells.

His contribution to the 2025 *RSC Chemical Biology* Emerging Investigators collection can be read at https://doi.org/10.1039/D5CB00247H.

